# Socioeconomic impact of artificial intelligence–driven point-of-care testing devices for liquid biopsy in the OncoCheck system

**DOI:** 10.1007/s10555-025-10281-3

**Published:** 2025-08-06

**Authors:** Sima Singh, Ada Raucci, Alessandra Glovi, Gabriella Iula, Luciano Mutti, Michelino De Laurentiis, Antonio Giordano, Stefano Cinti

**Affiliations:** 1 Department of Pharmacy, University of Naples ‘Federico II’, Via D. Montesano 49, 80131 Naples, Italy; 2 Scuola Superiore Meridionale, University of Naples “Federico II”, Naples, Italy; 3 Center for Biotechnology, College of Science and Technology, Sbarro Institute for Cancer Research and Molecular Medicine, Temple University, Philadelphia, PA USA; 4 Department of Biotechnological and Applied Clinical Sciences, University of L’Aquila, L’Aquila, Italy; 5 Department of Breast and Thoracic Oncology, Istituto Nazionale Tumori IRCCS “Fondazione G. Pascale”, Naples, Italy; 6 Department of Medical Biotechnologies, University of Siena, 53100 Siena, Italy

**Keywords:** Cancer inequities, OncoCheck, Liquid biopsy, Point-of-care testing, Artificial intelligence in cancer

## Abstract

Cancer disparities in low- and middle-income countries (LMICs) persist because of socioeconomic inequalities and limited access to screening infrastructure, which requires equitable diagnostic solutions. As researchers, we need to develop interventions which mirror successful strategies from high-income countries (HICs) to address mortality inequalities. Routine cancer diagnosis functions as a fundamental element of effective management yet remains unavailable to numerous populations in LMICs. This review proposes the conceptual “OncoCheck” model, which combines the terms Oncology “Onco” and Screening “Check” as an integrated approach to early cancer detection. It provides a theoretically sound practical approach that combines liquid biopsy with point-of-care testing (POCT) and artificial intelligence (AI) to achieve high-sensitivity diagnostics in resource-limited settings without requiring advanced infrastructure. The review advocates OncoCheck as a promising and practical cancer screening solution which shows potential to increase accessibility and decrease costs while improving survival rates through early detection. Moving beyond technical specifications, the manuscript assesses its socioeconomic impact, showing reduced medical costs and improved treatment outcomes. The paper describes its implementation framework together with a validation strategy and performance benchmarks. The analysis further focuses on the implementation barriers like algorithmic bias mitigation, infrastructure limitations, and ethical AI deployment. The OncoCheck system delivers equitable cancer care by implementing a hospital-at-home model which functions with real-world health systems.

**Graphical Abstract:**

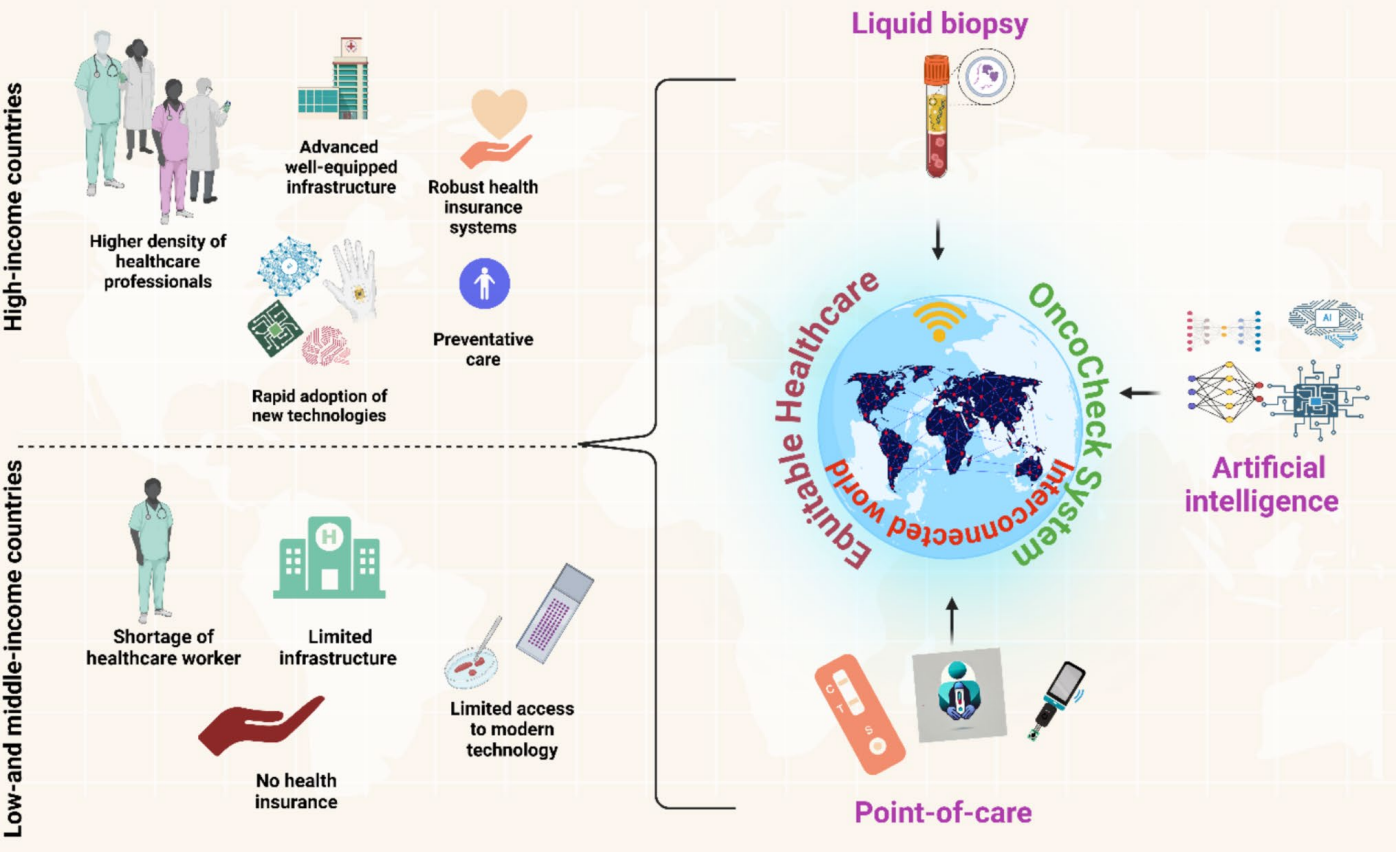

## From discovery to precision of cancer diagnostics

**Fig. 1:**
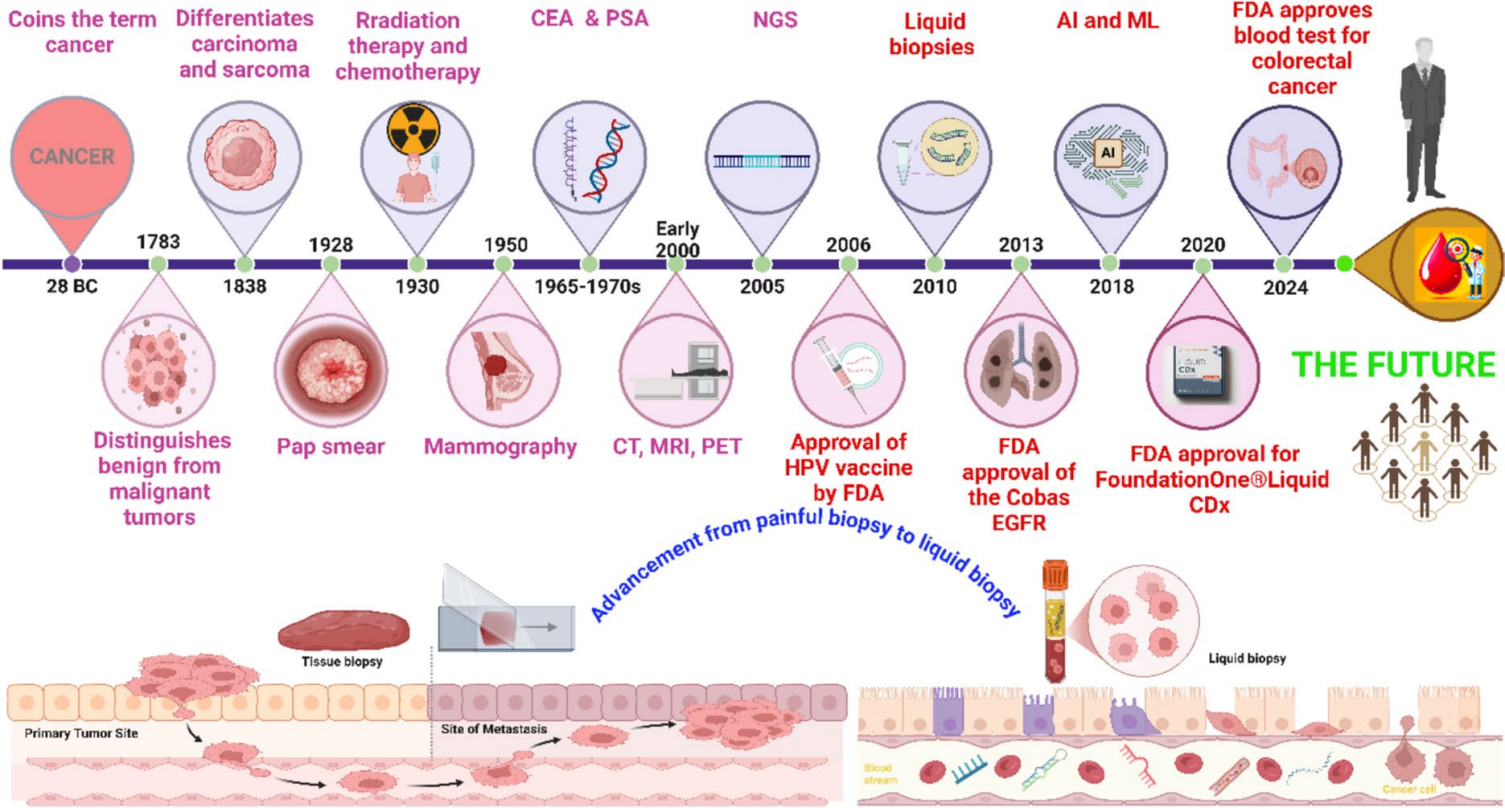
Historical timeline of cancer detection from traditional to AI-driven screening. The timeline focuses on the continuous development of technologies for personalized cancer management. The figure is created using BioRender

Cancer detection evolved from Celsus in 28 B.C. [1], benign-malignant distinction (eighteenth century) [2], carcinoma-sarcoma classification (1838) [3], the Pap smear (1930) [4], mammography (1950s) [5], to tumor markers like carcinoembryonic antigen (CEA) [6], and prostate-specific antigen (PSA) in 1965–1970 [7] providing new tools for cancer detection and monitoring. The twenty-first century has seen the rise of genetic profiling with next-generation sequencing (NGS) and liquid biopsy [8]. Liquid biopsy facilitates the comprehensive analysis of both deoxyribonucleic acid (DNA) and ribonucleic acid (RNA), providing deeper insights into the genetic and transcriptomic complexities of cancer cells [9]. It is exemplified by the Guardant360® CDx, an FDA-approved liquid biopsy test used as a companion diagnostic for *Kirsten Rat Sarcoma Viral Oncogene* (*KRAS p.G12C)* mutation detection in non-small cell lung cancer (NSCLC). It has demonstrated clinical validity for identifying patients eligible for sotorasib therapy, with high concordance to tissue-based testing [10]. Recently, artificial intelligence (AI)-driven machine learning (ML) refines diagnostics for personalized cancer care tailored to patient and tumor genetics [11]. Over time, cancer diagnostics have progressed significantly, as depicted in Fig. 1.

Despite advancements in AI and ML, access to cancer detection remains unequal worldwide. In low- and middle-income countries (LMICs), limited healthcare resources and late-stage diagnoses contribute to mortality rates as high as 70%, significantly higher than high-income countries (HICs) [12]. The mortality-to-incidence ratio (MIR) in HICs ranges from 0.36 to 0.48, indicating better survival outcomes due to improved healthcare access, while in LMICs, this ratio is 0.66 to 0.70, reflecting poorer access to early diagnosis and treatment. As a result, economic disparities between income levels worldwide are further exaggerated [13].

Cancer care disparities are driven by poverty, healthcare access barriers, and workforce shortages. For instance, one-third of countries lack access to radiotherapy, and sub-Saharan Africa has just 0.2 physicians per 1000 people compared to 3.7 per 1000 in high-income European nations [14]. Limited healthcare facilities hinder routine check-ups and early diagnosis. Unfortunately, cancer is often diagnosed at a more advanced stage, which results in increased treatment costs and decreased survival chances [15]. Scaling up screening and early-stage diagnosis in LMICs requires sustained financial support and supportive government policies. Implementing mobile point-of-care testing (POCT) units, decentralized diagnostic centers, and telemedicine-based consultations can improve access in remote and underserved regions. Studies indicate that treating late-stage cancers is 2 to 4 times more expensive than early-stage interventions. The World Health Organization (WHO) reports that early detection leads to a 50% reduction in total cancer treatment expenses [16]. The implementation of early detection techniques presents a solution to address resource or physician availability constraints in LMICs.

To this, liquid biopsy provides non-invasive detection of tumor heterogeneity, growth kinetics, and treatment resistance detection from a blood samples which serve as a minimally invasive alternative to traditional biopsies [17]. It contains various components such as RNA, DNA, circulating tumor cells (CTCs), and extracellular vesicles/exosomes [18]. The system enables cancer detection/monitoring by using easily obtainable fluids including blood, urine, and cerebrospinal fluid [17]^.^ The traditional lab-dependent methods including NGS, chromatography, and flow cytometry require infrastructure and expertise which restrict their application in remote or underserved regions [19].

POCT integration with liquid biopsy technology allows for faster and more accessible diagnosis because it eliminates the need of centralized laboratory testing [20]. The interpretation of data from liquid biopsy and POCT early detection methods becomes challenging in LMICs because of their limited analytical capabilities. AI-driven automated diagnostic models can enhance diagnostic accuracy, treatment customization, and patient outcomes [21–23]. A unified, accessible diagnostic platform is crucial to bridge the gap between early detection needs and healthcare limitations in LMICs.

**Fig. 2:**
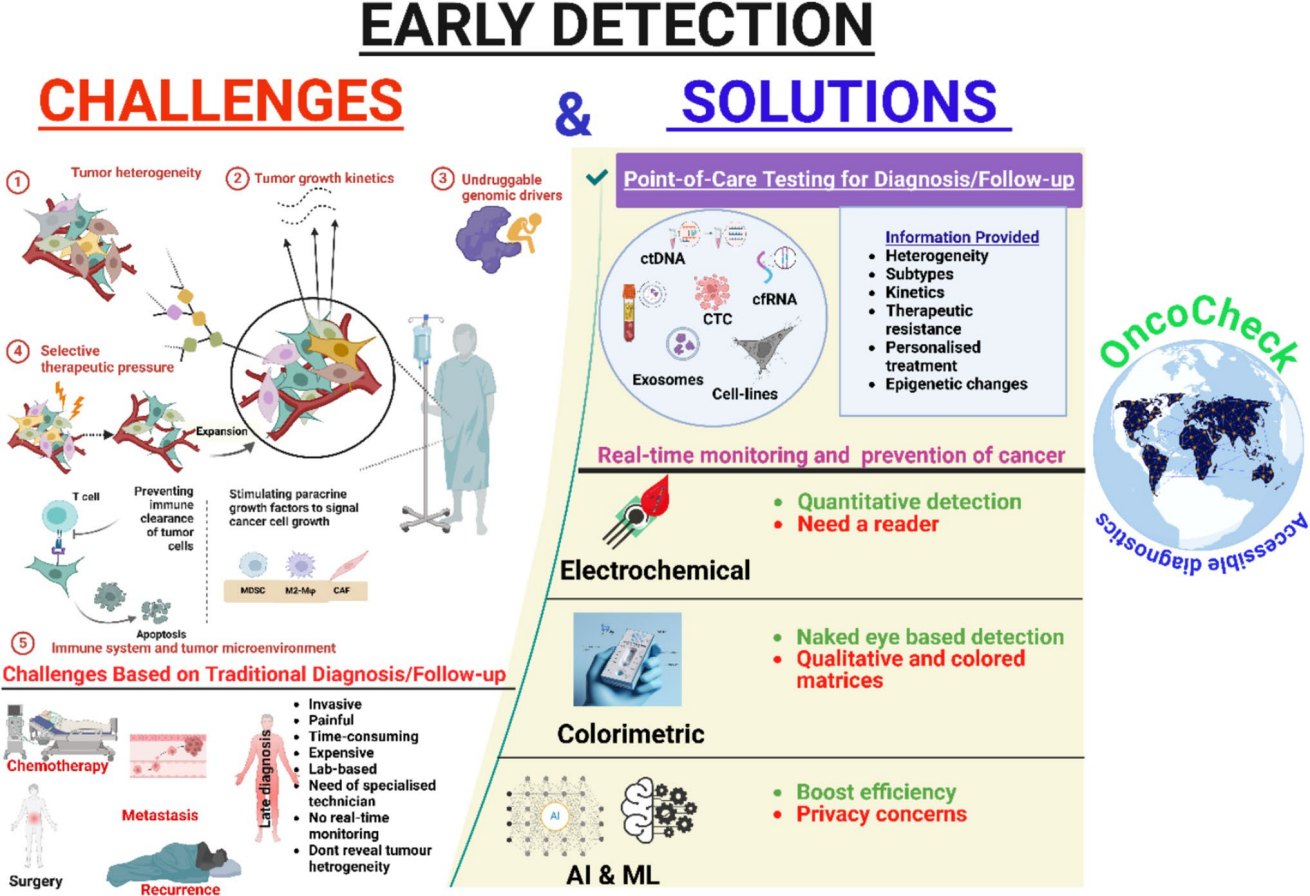
Illustration of the challenges and solutions associated with early cancer detection and treatment. The first part of the image highlights the existing challenges based on pathological conditions and conventional techniques. The second part proposes a liquid biopsy, POCT, and AI-integrated system named “OncoCheck” to address these challenges. The OncoCheck platform aims to ensure timely and equitable accessible diagnostics for all. The figure is created using BioRender

A diagnostic system that integrates liquid biopsy with POCT and AI functions as a solution to address long-standing problems in LMICs. The integration of these technologies creates a promising answer to the persistent obstacles in the path of cancer detection. The combined technologies enable fast and sensitive minimally invasive diagnostic capabilities which demonstrate great promise for worldwide healthcare needs. To represent this integrated approach, we introduce “OncoCheck” as a conceptual framework guiding this review. The term OncoCheck is derived from “Onco” for cancer and “Check” for screening, emphasizing its early detection focus. The diagnostic process starts with blood or body fluid sample collection followed by POCT-based biomarker assessment. AI models analyze results to detect genetic, epigenetic, and protein-based markers supporting both local and cloud-based decision systems to address infrastructure challenges. The goal is to establish a budget-friendly, non-invasive diagnostic solution that replaces conventional invasive medical procedures. Figure 2 outlines the conceptual model of OncoCheck and its potential to improve early cancer screening in resource-limited environments.

## Path to balance in cancer management across the globe

Achieving balance and bringing equilibrium throughout the world is not simple and cannot be achieved instantly because of existing disparities in cancer diagnostics and treatment access between HICs and LMICs. The root cause of disparity depends on many factors, starting from the economic crisis and basic infrastructure to competing health priorities. The existing healthcare facilities in LMICs have limited health system capacities and competing health priorities compared to HICs. Healthcare disparities arise from economic crises, infrastructure limitations, and competing health priorities [24]. For example, breast cancer survival rates range from 82% in Europe to 46% in Uganda, below 39% in Algeria, and in The Gambia, it is as low as 12% [25].

Limited availability of advanced diagnostic tools for early detection and treatment infrastructure restricts access to targeted medications [26]. Access to early cancer detection technologies is one of the major barriers to reaching this balance. Due to the unavailability of screening programs and affordable diagnostic tools, cancer is often diagnosed at an advanced stage. For example, the African continent has only 5% of the world’s cancer care resources available [27] in spite of 5% of the global total [28]. In LMICs, there are typically 0–2 megavoltage radiotherapy machines available for every 1000 patients in need of radiotherapy treatment, whereas in HIC, the ratio is usually between 3 and 7 machines per 1000 patients. Similarly, access to cancer treatments such as targeted medications, like trastuzumab, is also limited in LMICs compared to HIC [29].

**Fig. 3:**
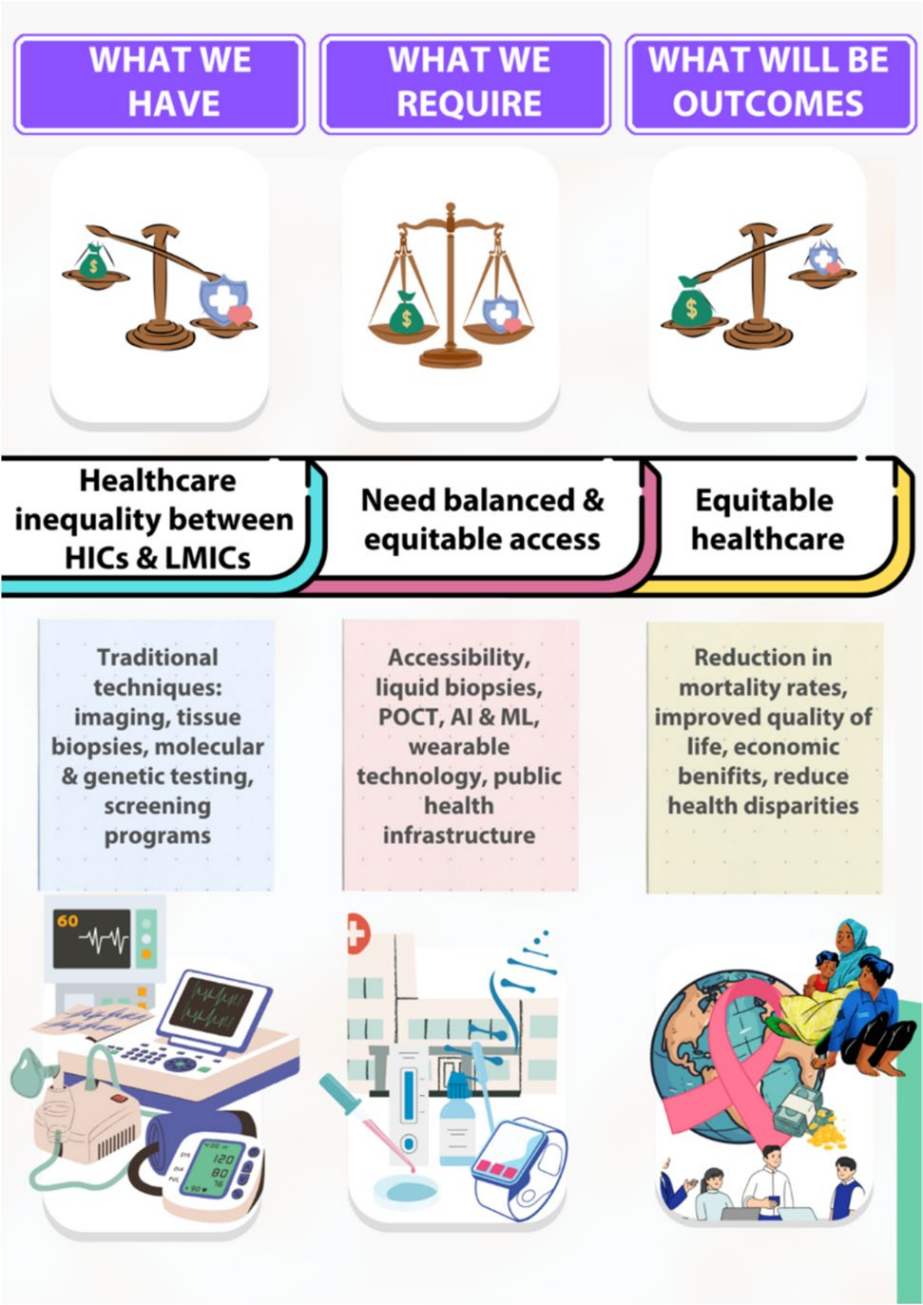
Path to balance equilibrium in cancer management across the globe. This image illustrates the existing inequality between HICs and LMICs, highlighting the need for equitable access to advanced technologies like liquid biopsies, POCT, AI, and wearable devices. Further, the outcome of implementing these changes will bring equitable healthcare

Although variations in disease disparities have been documented for decades, health equity has not been achieved to the greatest extent. Nevertheless, improvements in the healthcare infrastructure, the use of cost-effective diagnostic technologies, and the adaptation of therapeutic approaches have not been sufficient to ensure proper utilization. Figure 3 summarizes the current global disparities in cancer diagnostics and care, emphasizing the need for scalable and affordable solutions.

This gap stresses the importance of innovative and easily accessible, affordable, and decentralized diagnostic solutions at a cost-effective price that can be deployed in resource-limited settings. To overcome these inequalities, OncoCheck, which will provide equitable access to cancer diagnostics, can help reduce mortality rates, enhance quality of life, and decrease healthcare expenditures. The accuracy of the OncoCheck technology-assisted diagnostic system will be further enhanced, and data is easier to interpret and exchange, potentially for remote consultations, to make early cancer detection more available in the underserved regions. By bringing technology closer to underserved populations, we can reduce disparities in cancer outcomes and contribute to more equitable global healthcare settings for accessible diagnostics. This will improve the socio-economic conditions and build up the social cohesion of society by proposing fair and impartial accessibility across the globe for cancer detection.

Once we balance the equilibrium of “what we have” and “what we require” through the adaptation of OncoCheck, it will bring an equitable healthcare society that can diminish the existing disparities, cultivating a more just and inclusive society. The vision of OncoCheck is to bring healthcare communities and researchers closer to lay the foundation for the development of an equitable socio-economic society towards global health equity. Ultimately, achieving equilibrium can reduce treatment costs and improve the quality of life.

## Importance of adopting liquid biopsy, POCT and AI integrated system for cancer detection

However, for many patients, particularly of LMICs, out-of-pocket costs are still a big problem with regard to on-time cancer screening and treatment. It has been found that liquid biopsy is a more economical option than the regular screening methods such as magnetic resonance imaging (MRI) and traditional tissue biopsies, as the cost of screening patients is low. This benefit is highlighted by comparative studies in breast cancer detection; MR mammography (MRM) costs $6081/test [30], whereas the cost of liquid biopsy is between $149 and $187 [31]. Another study by Malczewska and colleagues found that a reliable biomarker could reduce the cost of detection in the USA by 50%. Current imaging expenditures range from $6000 to $24,000 per year. So, even if a single case could be prevented by a blood test, the annual savings would be between $200 and $500 million [32]. This comparative data demonstrates the significant impact of incorporating liquid biopsy into clinical practice to save time and cost.

Besides cost, however, selection and the precision of biomarkers are also important for enhancing disease conditions for which timely intervention or adjustments in treatment plans may have a significant impact on survival and quality of life [33]. The presence of *Estrogen receptor 1 (ESR1)* mutations in hormone receptor–positive breast cancer is associated with resistance to hormonal therapies [34]. The exosomal miRNAs have emerged as potential biomarkers for distinguishing pancreatic cancer from benign pancreatic conditions [35]. The prospect of being able to detect mutant genes at an early stage through blood in real time will greatly benefit clinicians in adjusting treatment regimens. It is a highly safe and user-friendly choice for personalized cancer care [36]. This increasing understanding of biomarker-directed disease monitoring not only helps in treatment personalization but also helps in detecting cancer at an early and premature stage. Scientific and technological advancements are evolving, and biomarkers, genomics, and emerging technologies are beginning to redefine the early cancer detection landscape through a simple blood test using liquid biopsy technology [37]. It offers a viable alternative to centralized lab-based methods [38].

Recent advancements in liquid biopsy have enabled early cancer detection through the identification of ctDNA and other biomarkers in asymptomatic individuals. Advances in ctDNA and other biomarkers have significantly improved early cancer detection, particularly through validated multicancer early detection (MCED) tests such as Galleri® (GRAIL) [39], CancerSEEK [40], and the SPOT-MAS test (K-DETEK study, ClinicalTrials.gov ID: NCT05227261), which can identify multiple cancers in asymptomatic individuals [41]. The DETECT-A study has further validated the feasibility of blood-based screening [42]. Additionally, cell-free DNA (cfDNA)-based tests have demonstrated high specificity and sensitivity in single-cancer detection, such as a colorectal cancer screening assay (83% sensitivity, 90% specificity) [43] and a cfDNA fragmentomic assay for early-stage non-small cell lung cancer (NSCLC) (83.2% sensitivity for Stage I tumors, 85.0% for tumors < 1 cm) [44]. While some liquid biopsy assays focus on early detection, others serve as comprehensive genomic profiling (CGP) tools for treatment guidance. FoundationOne® Liquid CDx, a CGP assay analyzing 324 genes, has been validated across > 7500 tests and 30,000 unique variants in solid tumors, making it a critical tool for identifying actionable mutations in advanced cancers and guiding targeted therapy [45]. Similarly, HelioLiver for hepatocellular carcinoma [46] and other single-cancer detection assays like the Lung-CLiP model for lung cancer [47] have undergone rigorous technical evaluation to enhance screening accuracy.

These exemplify the potential of liquid biopsy tests for high sensitivity and specificity. However, the lack of standardized protocols for sample collection and processing leads to inconsistencies in results, limiting its clinical adoption and also increasing the time and cost [48, 49]. However, widespread adoption of this technique is also not feasible for routine diagnostics; to overcome this, the adoption of integrated liquid biopsy and POCT technologies is transforming cancer detection and allowing patients and clinicians to track treatment responses or detect early signs of relapse with ease and precision [50, 51]. The FDA has also approved several POCT liquid biopsy cancer detection tests. In 2024, the agency approved Guardant Health’s Shield Test, a blood-based screening tool for colorectal cancer that uses blood-based screening by looking for tumor DNA in the bloodstream, a non-invasive alternative to colonoscopies [52]. These novel POCT liquid biopsy tests may provide faster, non-invasive cancer screening with earlier and more equitable detection.

So, liquid biopsy and POCT are two rapidly emerging technologies in oncology that offer distinct benefits individually. However, these technologies may not reach their full potential when used alone due to their limitations. Combining the strengths of both technologies and integrating them into a single platform can improve patient outcomes by enabling more timely and precise interventions. In recent years, the adoption of liquid biopsy and POCT technologies has revolutionized the field of oncology management, moving from a model of decentralized laboratory testing to bringing diagnostic capabilities directly to patients for timely detection and prevention [53]. Advanced transducer platforms of electrochemical (for glucometers) and optical (for pregnancy/COVID-19 tests) methods coupled with microfluidic biosensors can integrate liquid biopsy into the POCT platform, boosting the precision and specificity of detection [54]. Each method offers advantages and disadvantages. While electrochemical methods necessitate a portable reader and provide high sensitivity and quantitative/semi-quantitative detection, they remain unaffected by colored or turbid samples. On the other hand, colorimetric methods, despite their simplicity, enable visual inspection with the naked eye. However, the color of the matrix can impact their sensitivity [55].

Electrochemical biosensors facilitate the identification of biomarkers in body fluids such as blood, urine, and saliva. The unique sensors are suitable for developing countries because they can be deployed by portable devices. We can understand the wide application of electrochemical biosensors by discussing some existing examples. For example, Zhang and colleagues have developed an advanced electrochemical liquid biopsy (ELB) method using a hybrid ZIF-90-ZnO-MoS2 nanohybrid. This is a new technique that allows for direct identification of this cancer from blood samples. The developed sensing system can directly sort and process multiple signals, testing its high technical achievements [56]. The University of California, San Diego, used electrochemical impedance spectroscopy (EIS) and square-wave voltammetry (SWV) to find soluble KIT, a biomarker for cancer, in blood samples. The LOD was 1 pg/mL, and the dynamic range was 10 to 100 ng/mL. The proposed aptasensor has a vast array of applications in cancer therapy monitoring and diagnostics (theranostics), providing a continuous or intermittent approach [57]. A paper-based electrochemical platform was developed to detect miRNA with high sensitivity levels. The platform can identify miRNA at concentrations in the nanomolar range. It has the potential to be a valuable addition to cost-effective and widely accessible cancer screening methods [58].

Lateral flow assay (LFA) is a promising user-friendly diagnostic option that has gained popularity due to its accessibility, simplicity, affordability, and quick results without requiring equipment [59]. LFA signals can be amplified using readers, which stimulate nanoparticle labels with external stimuli like laser light, electric potential, or magnetic field. Sensitive optical, electrical, and magnetic sensors detect these amplified signals, enhancing detection sensitivity by several orders of magnitude over traditional visual readouts [60]. However, the choice between the diagnostic techniques also depends on both the economic and clinical aspects. For example, some cancer detection based on the LFA technique is remarkably affordable and available at $10. The affordability price is complementary in comparison to conventional diagnostic approaches, like imaging methods, tissue biopsies, and enzyme-linked immunosorbent assay (ELISA) tests. Typical diagnostic tools such as imaging or tissue biopsy cost hundreds or even thousands of dollars [61]. Prostate cancer biopsy and PSA tests cost $315 and $19, respectively, according to reports [62].

Despite being a highly valuable technique for cancer detection due to its high sensitivity and specificity, the high cost of ELISA limits its wide application. The high cost of conducting ELISA tests for detecting cancer depends on the cost of reagents and equipment maintenance expenses, along with labor costs and overhead charges. For example, Abcam offers quality commercial ELISA kits for cancer biomarkers like PSA or CEA that can range from $100 to $1000 per kit, depending on the number of tests it can perform [63]. In the same way, the limited availability or scarcity of imaging machines per million people leads to a long waiting time before they can undergo cancer screening. A delay in diagnosis results in a more advanced stage of cancer detection, which further delays the implementation of therapies.

So, in these scenarios, LFA-based POCT is an alternative practical solution. For this purpose, a commercial LFA kit such as the OncoQuick® diagnostic test is the most effective solution, delivering results within a few minutes using a blood sample. This makes it a valuable tool for early cancer detection in resource-limited settings [64]. Apart from this, few researchers are actively involved in the development of LFA-based platforms for cancer detection and management. Kim and colleagues proposed DNA barcode-based detection of exosomal microRNAs using nucleic acid LFA for colorectal cancer diagnosis. In human plasma samples, one strip simultaneously detects multiple exosomal miRNAs with a sensitivity of 95% and a specificity of 100% [65].

The development of POCT systems that combine microfluidics with electrochemical and LFA detection techniques presents a promising middle ground for liquid biopsies-based cancer detection. Microfluidic-based assay examples offer precise fluid manipulation, reduced sample and reagent usage, and a high level of integration. These advantages contribute to improved efficiency and accuracy in testing [66]. This synergy provides advantages by mainly improving the accuracy and effectiveness of diagnostic processes. Universities and laboratories are striving to create cost-effective versions of microfluidic tests for use in developing nations due to their high cost. The CTC chip (invented at MGH, Boston, MA) is a microfluidic device consisting of geometrically arranged antibody-coated microposts to improve cell adhesion. The CTC-iChip, which uses both microfluidic and immunomagnetic technologies, has demonstrated a higher sensitivity to CTC identification compared to CellSearch [67]. Qian and his colleagues suggested a simple, quick, and portable way to separate exosomes and find exosomal microRNA in a microfluidic device based on agarose. This device has a low LOD of 10^3^ exosomes [68].

These discussions highlight the technological advancements in liquid biopsy-based cancer detection and monitoring using electrochemical and optical biosensors along with microfluidic technology. It discusses the progression from laboratory research innovations to practical application of the innovations, with emphasis on the increasing role of non-invasive, rapid, and easily performed cancer diagnostics in enhancing early detection and individualized treatment plans. Table 1 summarizes a practical perspective by listing the currently FDA-approved diagnostic and monitoring kits in clinical use. These kits are based on various biomarkers such as protein, genetic, and cellular.

**Table 1:** FDA-approved cancer diagnostic and monitoring kits using liquid biopsy and POCT technologies

Device/company	Assessment condition	Cancer type(s)	Molecular target	Sample type	Sensitivity	Specificity	Reference
Nodify Lung® (Biodesix)	Risk stratification of indeterminate pulmonary nodules (IPNs) to aid early detection and guide management	NSCLC	Autoantibodies against 7 lung cancer-associated antigens (*p53, NY-ESO-1, MAGE A1, GBU4-5, CAGE, HuD, SOX2*)	Blood (plasma)	16% (95% CI: 12–22%)	90% (95% CI: 85–93%)	[69]
Guardant360 CDx	Companion diagnostic for targeted therapy selection	NSCLC, others	cfDNA mutations (*EGFR*, *KRAS*)	Blood	88.9	High	[10]
Dxcover Brain Cancer Liquid Biopsy (Sensitivity Tuned Model)	Early detection of brain tumors (prior to imaging confirmation)	Glioblastoma, Astrocytoma, Metastatic brain tumors	Spectroscopic biochemical signature (FTIR spectroscopy of serum)	Blood (serum)	96% (for brain tumors, across WHO grades I–IV)	45%	[33] [70]
Galleri (GRAIL)	Multi-cancer early detection	> 50 types	cfDNA methylation patterns	Blood	NA	98·7–99·4%	[39]
FoundationOne Liquid CDx	Comprehensive genomic profiling	Solid tumors	*cfDNA* (324 genes)	Blood	High	High	[45]
CancerSEEK	Early detection of multiple cancers	Ovary, liver, pancreas, etc	cfDNA mutations + protein biomarkers	Blood	69–98%	> 99%	[71]
Cologuard	Colorectal cancer screening	Colorectal	Stool DNA + hemoglobin	Stool/hemoglobin	92.3%	86.6%	[72]
NAFLD Polygenic Risk Score (NAFLD-PRS) – Research Prototype	Genetic predisposition evaluation for early HCC screening in lean NAFLD individuals	Hepatocellular Carcinoma (HCC)	SNPs in *PNPLA3, GCKR,* GATAD2A (East Asian PRS)	Blood (via genomic DNA)	33% at optimal cutoff (AUC: 0.57)	74% at optimal cutoff (AUC: 0.57)	[73]
Hepatic Fat Content PRS (HFC-PRS) – Research Prototype	Genetic score to stratify hepatic fat-associated HCC risk	HCC	SNPs in PNPLA3, *GCKR*, *TM6SF2*, *MBOAT7* (European PRS)	Blood (via genomic DNA)	33% at optimal cutoff (AUC: 0.59)	87% at optimal cutoff (AUC: 0.59)	[73]
AdnaTest ProstateCancerPanel *AR-V7* (QIAGEN) (JHU & Epic Assays)	Predicts resistance to hormone therapy (abiraterone/enzalutamide) in mCRPC	Metastatic Castration-Resistant Prostate Cancer (mCRPC)	*AR-V7* mRNA in CTCs (JHU assay) and nuclear *AR-V7* protein (Epic assay)	Blood	JHU Assay: 95% (analytical), 24% clinical positivity; Epic Assay: ~ 100% specificity, 9% clinical positivity	JHU: ~ 89% (*AR-V7* −); Epic: 100% (no PSA response in *AR-V7* +)	[74]
Shield™/Guardant Health	Non-invasive screening for colorectal cancer in average-risk adults aged 45–84	Colorectal Cancer (CRC)	ctDNA with genomic, methylation, and fragmentomic patterns	Blood (Plasma)	83.1% (overall CRC); 87.5% (Stage I–III); 13.2% (advanced precancerous lesions)	89.9% (for negative colonoscopy); 89.6% (for no advanced neoplasia)	[43]

While liquid biopsy and POCT have significantly improved the accessibility of cancer diagnostics, however, these tools alone are insufficient to address the complexities of both disease and data. AI enhances cancer diagnostics by interpreting complex clinical data, which leads to precise and optimal patient management [75]. The use of AI can be integrated further to revolutionize cancer diagnostics; however, the high costs, lack of digital infrastructure, and shortage of trained human resources in the healthcare system are limiting its application in low-resource environments. POCT devices, optimized by AI, give real-time on-site cancer detection without requiring centralized facilities and hence give instant results, even in remote and rural areas. A notable example is the Sangia POCT-based PSA test, Silver Amplified NeoGold ImmunoAssay (Sangia) Total PSA Test (OPKO Health Inc.) on Jan. 30, 2019, as the first POCT-based prostate cancer detection that delivers rapid AI-assisted prostate cancer diagnostics using only a small blood sample [76].

**Fig. 4:**
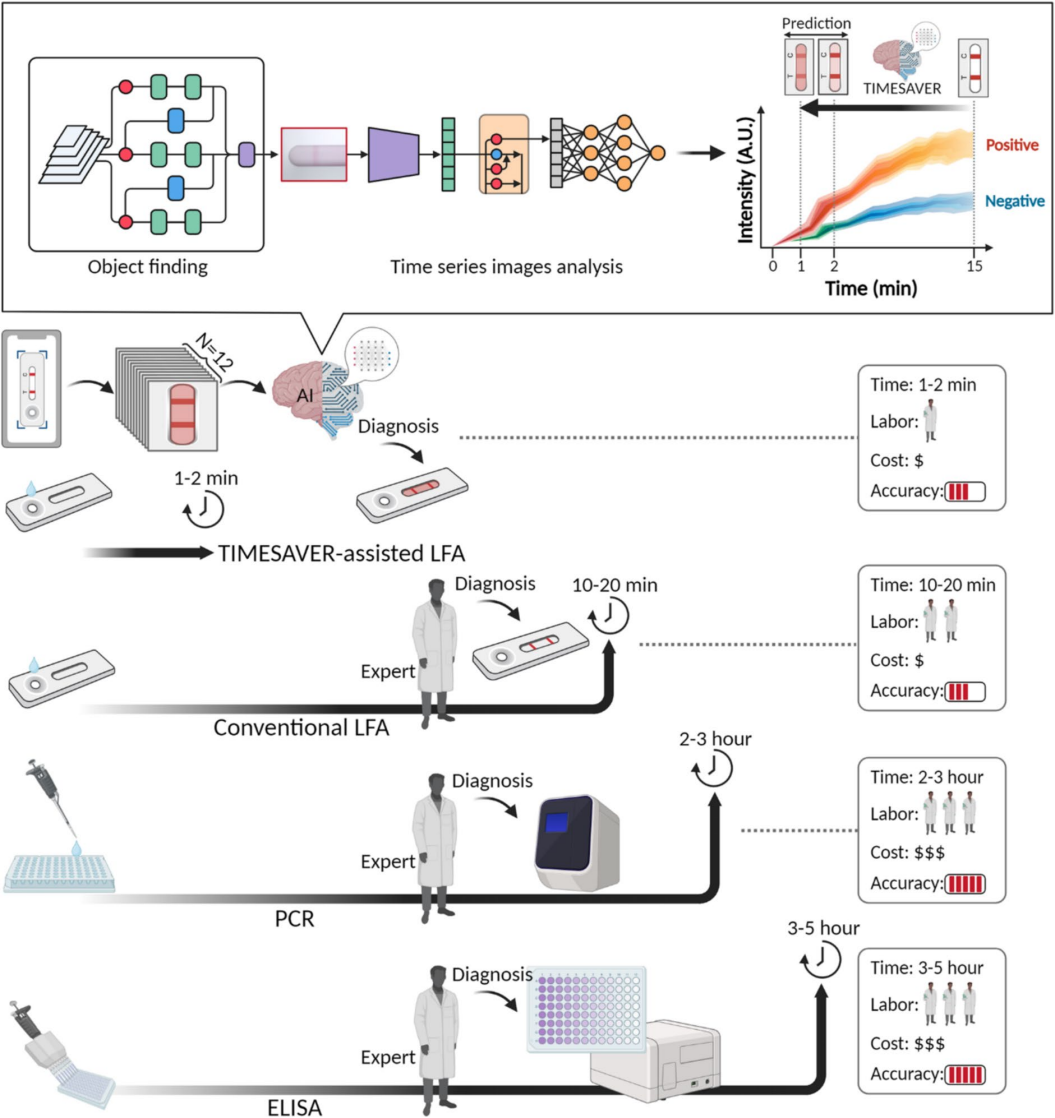
Comparison of diagnostic tools: TIMESAVER-assisted AI-Enhanced LFA vs. conventional methods [79]

Another transformative solution is cloud-based AI platforms that can help in remote cancer diagnosis by processing data in high-resource settings. Remote diagnosis of cancer is possible with the help of AI-driven image recognition models that analyze histopathology slides, overcoming the shortage of pathologists in LMICs [77, 78]. Moreover, the AI-enabled diagnostic smartphone applications can help to diagnose the disease at the patient’s home by providing self-screening with the help of AI-augmented LFAs. For instance, integration of AI increases the efficiency of technology like LFA, especially for POCT settings where speed, cost, and minimal labor are critical. By integrating AI for real-time interpretation, TIMESAVER offers a streamlined alternative to conventional methods, combining the accessibility of LFA with the precision and efficiency of AI-driven analysis. This system can provide analysis results within 1 to 2 min as opposed to LFAs that usually take around 10 to 20 min as shown in Fig. 4 [79].

AI algorithms must also be tailored for low-data environments, where access to high-quality datasets is limited. ML models trained on smaller, region-specific datasets can be deployed on low-power devices, reducing dependency on extensive computing infrastructure. AI is transforming the landscape of cancer detection by utilizing various approaches, such as ML. ML is a subset of AI that uses data to teach machines and demonstrates proficiency in interpreting medical data, such as imaging, genomics, and pathology slides with high precision [80]. For instance, ML was utilized for CTCs and ctDNA detection, for pinpointing cancer locations [81]. Furthermore, this information helps clinicians make treatment choices as per the patient’s requirements. So, with the integration of AI, patients can take charge of their health decisions as the healthcare system becomes more inclusive and accessible to everyone [82]. AI has promised to transform medical practice by utilizing various approaches, like microfluidic biosensors that use AI to detect ctDNA in a single blood drop, offering a cost-effective and minimally invasive alternative to traditional biopsy methods [83]. These AI-driven solutions have the potential to democratize cancer diagnostics, ensuring that early detection is not limited by geographical or economic barriers.

The literature has reported various studies on the use of AI/ML in early cancer diagnosis, like the lung cancer likelihood in plasma (referred to as the Lung-CLiP model). It demonstrates the application of ML technology for detection by analyzing genetic traits enabling non-invasive detection of lung cancer [84]. Recently, there has been a focus on developing nanosensors specifically designed for use in the field of medicine and health care applications, particularly for liquid biopsy systems. They show potential in pinpointing different biomarkers linked to gynecological cancers. This sensor could completely change the way medical tests are done by being placed inside patients’ body fluids and uterine cavities, offering a level of accuracy in detecting cancer at an early stage with the help of advanced ML programs that interpret the information gathered [85]. Furthermore, a ML approach combining support vector machine-recursive feature elimination (SVM-RFE) and artificial neural networks (ANN) was employed to develop a diagnostic model for the detection of microRNAs that differentiate between pancreatic and non-pancreatic cancer. The identified miRNA biomarkers showed high accuracy in distinguishing between normal and cancerous samples [86].

Beyond improving individual patient outcomes, integration of AI offers significant economic benefits. Marketing and cancer research firms have projected that the savings resulting from the use of AI technology in the healthcare industry in the USA could amount to around $52 billion in 2021. In order to enhance the effectiveness of AI’s role in advancing research for treating cancers, it is crucial to have access to sufficient data for refining ML and deep learning models [87].

### OncoCheck-conceptual system architecture and proposed validation pathway

System Implementation and Architecture: The OncoCheck system is designed as a tri-modular, integrative diagnostic platform that combines three critical components to enable rapid, decentralized cancer detection: (i) liquid biopsy, (ii) POCT biosensor platform, and (iii) AI-driven data interpretation.

The first module, the liquid biopsy chemistry component, uses blood or fluid samples to detect cancer biomarkers which include ctDNA, miRNAs, and proteins. The approach is minimally invasive and detects molecular alterations that are linked to tumors which enables early diagnosis and monitoring. The system simplifies sample processing and biomarker detection which supports precision oncology through fast and efficient analysis [88]. This module draws upon established techniques seen in commercial platforms like Guardant360’s ctDNA assays and research-grade exosome-based diagnostics, but with optimizations for low-resource settings [89].

The second module consists of a POCT biosensor platform designed for portability and ease of use. This component works on the latest electrochemical sensing and lateral flow technology to provide rapid equipment-free quantification of cancer biomarkers. Recent innovations have validated the platform’s ability to detect ctDNA and miRNAs with exceptional sensitivity and specificity, enabling robust early-stage cancer diagnostics while maintaining compatibility with resource-constrained settings [90]. The system draws its design from established diagnostic tools such as Cepheid’s portable nucleic acid tests and contemporary biosensor platforms. The system is designed to be cost-effective and diagnostically accurate. The interface requires minimal user input. This makes it ideal for environments with limited technical resources [91].

The third module integrates an AI-driven data interpretation engine to enhance diagnostic reliability. The system uses ML algorithms trained on curated datasets from sources like the Cancer Genome Atlas (TCGA) to analyze biomarker patterns and reduce diagnostic uncertainty [92]. The system follows a similar decision-support model as IBM Watson Oncology, but it is designed to process unprocessed biosensor data. The AI component functions as a vital risk stratification tool because it operates in areas with restricted specialist consultation access [93].

The OncoCheck system requires a systematic validation process to measure how well the system performs against gold-standard assays through sensitivity and specificity tests. The first phase of this phase involves testing spiked samples and clinical specimens from the past to establish baseline performance metrics [94]. The next step would involve clinical feasibility studies in decentralized, real-world settings to assess operational usability and effectiveness, similar to validation protocols used in point-of-care technologies like the Cepheid GeneXpert [95]. The last step requires regulatory approval through demonstrating conformity with existing *in vitro* diagnostic standards as mandated by the *In Vitro* Diagnostic Regulation (IVDR) which requires conformity assessment by notified bodies [96].

The system needs real-world implementation through partnerships with global health organizations to deploy it in LMICs where its impact on early cancer detection would be most significant. The system would demonstrate its potential to close diagnostic gaps in underserved populations through its alignment with PATH’s cancer screening programs [97]. The system’s utility in precision oncology would advance through future iterations that either expand its biomarker panel or enable wearable sensor compatibility.

The OncoCheck conceptual model demonstrates a comprehensive framework for transforming cancer diagnostics into a single integrated system. The framework demonstrates how new technologies will increase detection speed and enhance treatment results especially in areas with limited access. The design incorporates established methods while maintaining its unique focus on decentralization and practical implementation. OncoCheck offers a clear path to convert innovative concepts into practical diagnostic solutions which can solve worldwide early cancer detection challenges.

However, the practical implementation of OncoCheck in diverse LMICs presents several challenges. The scalability of AI-powered diagnostic tools faces limitations because of variations in infrastructure which include unreliable electricity, limited internet connectivity, and inconsistent supply chains. The adoption of telemedicine at the community level faces challenges due to a shortage of trained healthcare workers and limited digital literacy among community members. These practical constraints must be addressed through locally adapted deployment strategies, phased pilot programs, and government-supported frameworks to ensure equitable adoption.

### Socioeconomic impact analysis of the proposed OncoCheck concept

Cancer creates major economic losses in LMICs which affect both household financial stability and national productivity. The regions experience delayed diagnosis which results in both high treatment expenses and devastating economic effects on families. The total burden of cancer includes both direct medical costs of treatment and indirect costs from lost income because of cancer-related disability and death. The OncoCheck model serves as a theoretical solution to tackle these challenges by enabling early cancer detection through decentralized diagnostics.

Evaluating the potential socioeconomic impact of decentralized cancer diagnostics requires careful analysis of published cost data. GRAIL reports estimate early detection could cut cancer treatment costs by up to 17%, translating to $26 billion in savings [98]. The costs of treatment become significantly higher when cancer advances to its later stages. The total cost of breast cancer treatment in LMICs varies widely, ranging from $195 to $11,866, depending largely on the healthcare system’s capacity and the stage at diagnosis, as reported by Yeong et al. The total healthcare expenditure includes direct medical costs which make up between 50 and 80% of the total amount, while inpatient care together with surgical procedures, radiation therapy, and medications represent the main cost factors [99].

Further insight into the economic impact of cancer in LMICs can be derived from regional studies. Daroudi et al. (2015) estimated that the total economic burden of breast cancer in Iran was approximately $947.4 million in 2010. Notably, 77% of this burden was attributed to productivity losses arising from premature mortality and disability, while only 18.56% was due to direct medical costs. Among the direct costs, chemotherapy represented the most significant expense at $76.7 million, with trastuzumab prescriptions alone accounting for 41% of chemotherapy spending ($31.5 million) [100]. Similarly, head and neck cancers contribute substantial economic losses in LMICs. A study from Brazil reported that head and neck cancers imposed an annual economic burden of $534 million, with direct medical costs representing 58% and indirect productivity losses contributing 42% to the total [101].

These findings consistently underscore that delayed diagnosis not only elevates medical expenditures but also generates significant national economic losses due to workforce attrition. To mitigate these burdens, early diagnosis at the primary care level must be prioritized, coupled with strategic investments in low-cost diagnostic innovations capable of detecting malignancies at an earlier, more treatable stage. Future policy frameworks must integrate early detection within broader socioeconomic planning to prevent long-term health system strain and national economic loss.

Beyond direct medical costs, cancer care imposes a heavy financial burden through catastrophic health expenditures (CHE), productivity losses, and non-medical expenses. The ASEAN Cost in Oncology (ACTION) study found that within 12 months of diagnosis, 48% of patients experienced CHE and 29% died, with low-income patients facing nearly six times higher odds of financial catastrophe compared to wealthier groups [102]. Non-medical costs such as travel can account for up to 25% of the total patient burden, with average transportation costs reaching $50–$100 per hospital visit [103]. Productivity losses alone contributed to over 40% of the total cancer-related economic burden globally [13].

These costs are prohibitive for rural populations, leading to delayed or foregone diagnosis. Deployment of community-based POCT would significantly eliminate transportation-related financial barriers. National policies should mandate the integration of mobile screening units into rural health delivery systems, ensuring geographic equity in cancer diagnosis.

Strategic deployment of affordable, AI-enabled diagnostic platforms can replicate this cost-saving phenomenon in resource-constrained settings. Health technology assessments must prioritize early diagnostic interventions not solely on clinical effectiveness but also on national economic return. Thus, the full economic analysis underscores the urgency for systemic adoption of decentralized early diagnostic systems such as the proposed OncoCheck model. Policymakers must move beyond treatment-centric paradigms and focus on early diagnosis as an essential investment into national economic stability and equitable healthcare outcomes.

## Concluding remarks and future directions

OncoCheck introduces liquid biopsy, POCT, and AI to precise, accessible, and early cancer detection. OncoCheck enables healthcare providers to detect cancer through cancer markers using liquid biopsy with minimal patient discomfort, and it is proposed as a sustainable and economically viable cancer screening model. It reduces hospital dependency and promotes POCT cancer detection, which reduces the burden of health facilities. Also, AI-driven diagnostics improve the effectiveness of resource utilization, for proper and timely decision-making in cancer management.

The proposed OncoCheck system enables standard screening for cancer in remote areas. Integrating POCT provides the possibility that tests are easily accessible, and the outcome of the tests is provided quickly at the point of contact itself. This is very important in rendering medical assistance towards improving the outcomes in resource-limited areas. It further decreases the need for patients to travel long distances to specialized cancer centers. OncoCheck uses AI algorithms to improve healthcare diagnoses by identifying cancer signs with accuracy, simplifying complex data interpretation for LMIC areas, where shortages of trained specialists and diagnostic equipment limit access to cancer diagnostics. Thus, in the long-term, adaptation of OncoCheck technology will cut down on the overall cancer treatment costs as well as improve the survival rates significantly.

Despite its advantages, there are some limitations, such as outcomes being affected by tumor heterogeneity [104] and low biomarker levels in early-stage cancers [105], which may lead to reduced sensitivity and specificity. Furthermore, the presence of false positives and negatives is a challenge, which makes it necessary to validate the findings with traditional biopsy methods [106]. Another limitation is that the analysis is AI-driven and can introduce algorithmic biases. Moreover, if AI models are trained on genomic datasets that lack diversity, then disparities can occur in diagnostic accuracy [107, 108].

Another concern relates to algorithmic fairness and transparency. If AI models are trained primarily on datasets from HICs, their diagnostic accuracy may drop when applied to genetically or demographically different populations. This can inadvertently introduce healthcare disparities [109]. Additionally, many AI tools lack explainability, which limits trust among healthcare providers and patients. To mitigate these risks, future implementations must prioritize training models on diverse datasets, ensure interpretability of AI decisions, and establish protocols for human oversight to verify outputs in critical cases.

POCT devices driven by AI require large-scale genomic data and patient records. Unfortunately, using AI is not without strings; there are social and ethical challenges to human rights, security, and privacy [110]. Combining AI and ML into healthcare raises concerns about the principles of informed consent and transparency. Patients and healthcare providers must understand how these algorithms work to trust AI-based diagnoses. Keeping these trust relations requires making the decision-making processes transparent. Solving these problems will require worldwide collaboration to establish the standard AI processing rules as well as ensure fair representation of individuals in AI training datasets [111]. The different regulatory approvals across different countries are an issue for adopting this technology.

Furthermore, ethical and logistical concerns, including consent, privacy, data sovereignty, and long-term sustainability of cloud-based AI infrastructure, must be critically addressed before widespread implementation, especially in settings where regulatory oversight remains weak.

For successful integration of AI-driven diagnostics in cancer screening, it is important to have a well-structured policy framework that guarantees accessibility, affordability, and equity of the service especially in LMICs [112]. The use of AI in cancer detection requires the collaboration of governments, healthcare institutions, technology companies, and regulatory authorities. In cases of AI adoption, especially in developing countries, public–private partnerships (PPPs) can help subsidize AI adoption that can accelerate the development of cost-effective solutions [113, 114]. International regulatory agencies need to provide a uniformity of standards for AI-based diagnostics, including processes for thorough clinical validation and transparency in algorithmic decision-making. Genomic data-sharing initiatives should promote the development of AI-ready datasets that have diverse genomic profiles, and cloud-based AI models should be adopted to remotely process the data and reduce reliance on expensive local computing infrastructure [115]. Governments should invest in AI literacy programs for healthcare professionals, integrating AI training into medical education curricula to bridge the digital divide [116].

**Fig. 5:**
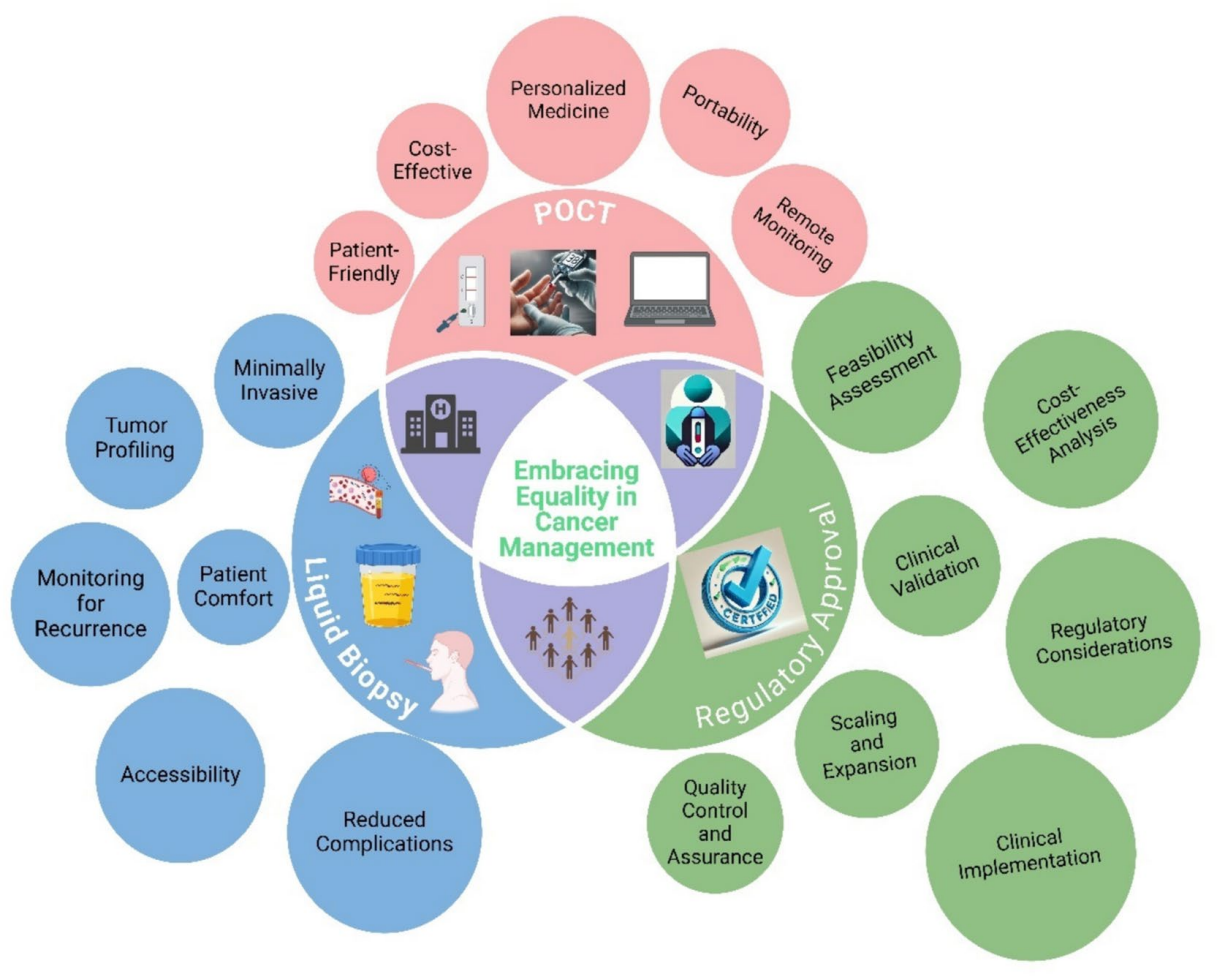
Framework for bridging the gap and bringing equitable cancer management through integration of liquid biopsy, POCT, and regulatory pathways

In the coming years, significant progress is expected in the field of cancer management due to advancements in technology. The future of cancer detection depends on integrating innovative diagnostic tools with equitable healthcare access. By focusing on the themes of this paper: promoting equality, fostering regulatory support, advancing POCT and liquid biopsy technologies, and ensuring standards, we are taking steps towards a future in healthcare where detecting cancer early and providing personalized treatment are available to everyone, irrespective of their socioeconomic status and geographic barriers as shown in Fig. 5.

This vision aligns with the broader goal of reducing global health disparities and establishing a resilient, inclusive healthcare system for the future. With this, we are closing our review with the quote of hope: “health should be a fundamental human right, not a privilege paid in technology or currency.”

## Data Availability

No datasets were generated or analysed during the current study.
